# Organic Chemistry in Its Application to Agriculture and Physiology

**Published:** 1841-04

**Authors:** 


### ART. VIII.

*Organic Chemistry in its application to Agriculture and Physiology.* By JUSTUS LIEBIG, Ph. D., Professor of Chemistry in the University of Giessen. Edited from the MSS. of the author by LYON PLAYFAIR, Ph. D.—*London,* 1840. 8vo, pp. 387.

*Traité de Chémie organique.* Par M. JUSTUS LIEBIG. *Introduction.* —*Paris,* 1840. 8vo, pp. 195.

THE two titles which we have given belong to one and the same book. An edition has also appeared simultaneously in the German language. The idea of writing the work, however, was suggested to the author in England. Professor Liebig visited this country in 1837, and was present at the meeting of the British Association at Liverpool, when he communicated the intelligence of the remarkable discovery made by himself and Wöhler, viz., the formation of allantoin, a constituent of the liquor amnii of the cow, in the laboratory, which constituted the second example of the production by purely chemical means of an animal product. Wöhler had previously formed urea by passing cyanic acid through ammonia, and had thus opened a field of almost unbounded discovery and of vast importance to the physiologist. At the meeting to which we have referred the author, who, although in the prime of life, has already been for some years in possession of a European reputation in connexion with the chemistry of organic matter, was requested, on the motion of Dr. Thomson, of Glasgow, to draw up a report, for the Association, on the state of organic chemistry. Liebig cordially assented to this serious call upon his time. The result is now before us. The object of the author in this elaborate report has not been to present to his readers a catalogue of isolated facts; but he has endeavoured as far as possible to apply the doctrine of organic chemistry so as to explain the apparently mysterious processes of vegetable and animal growth. To plant the seed in the barren soil, to see it sprout into a living thing, to observe it gradually increase in weight and size until it has obtained an altitude perhaps immense, and all without apparent motion or sound, is at once most wonderful and most mysterious. But to analyze the soil in which the seed is sown, to investigate the properties of its varied constituents, the water which falls from the clouds to moisten it, the atmosphere in which it is immersed, and all the elements which act upon its existence is, partly at least, to elevate the veil of mystery and reveal to us the works of the simple and noiseless machinery of nature.

Liebig divides his work into two parts. In the first he discusses the chemical processes in the nutrition of vegetables, and dwells especially upon the secondary causes of nourishment. The second division is devoted to the chemical processes of fermentation, decay, and putrefaction, by means of which he considers nutrition to be supplied from its primary sources, contagions and infections generated both in and out of the human body, and a perpetual succession of changes in the form of matter to be kept up. Matter thus assumes alternately inferior and beautiful forms, in such infinite variety and so worthy of admiration that " even custom cannot stale" them.

Many of the views of the author are so novel and of such extensive application, that it is somewhat difficult to afford a correct analysis of them in a short space. We shall endeavour, however, to take notice of the principal applications of them to theory. We follow in general the French edition, in which the various subjects are more readily found, from more convenient arrangement.

*Food of plants.* It is obvious from even a superficial survey that plants require food just as animals do, and that the sources of their nourishment must be the soil in which they are planted and the air in which their stems are immersed. Van Helmont had deduced from an experiment on a willow that rain-water alone was sufficient for the support of the plant, because when supplied with this kind of food, to all appearances alone, it increased from 5 to 164 lbs. Bergmann, however, showed that 1 lb. of rain-water contained 1 grain of earthy matter, which would amply suffice for contributing inorganic matter to the plant. Van Helmont also omitted in his calculation the substances absorbed from the atmosphere by the soil, and conveyed into the vessels of the plant; and, finally, though this is not the least important consideration, he forgot the nutritious power of the atmosphere.

When plants are examined by destructive analysis they afford carbon, nitrogen, and oxygen and hydrogen, in the proportions to form water. It will be convenient to consider the sources of each of these separately.

*Assimilation of carbon.* Every one is familiar with the fact that when we burn a piece of straw at a candle a portion is consumed, and what remains is charred or blackened at the edges. Few consider that this is a process of destructive analysis. The oxygen and hydrogen are driven off, and the carbon or charcoal—the main constituent of the organic substance—remains behind. From whence does the carbon derive its origin? Liebig endeavours to answer this according to the strictest rules of analytic reasoning. Physiologists have been in the habit of viewing the decomposing vegetable matter of soils, or *humus*, as one of the principal sources of the food of plants. This substance they conceive to be absorbed by the roots and thus conveyed into the proper assimilating vessels, where its carbon is abstracted. Humus is described by chemists as a brown matter, dissolving partially in alkalies. The portion which dissolves in alkalies has been termed *humic* acid; the insoluble part *humin*. A careful chemical examination of the substance, however, has demonstrated that it possesses no constant chemical constitution, and that as it is formed in the soil it is merely a vegetable matter in various stages of decay. Liebig, in showing the fallacy of the usual opinion respecting the influence of humus in the nutrition of plants, proves his point by a *reductio ad absurdum*. He admits, for the sake of argument, that humic acid is absorbed by plants in the form of that salt which contains the largest proportion of humic acid, viz., in the form of humate of lime, and then from the known quantity of the alkaline bases contained in the ashes of plants he calculates the amount of humic acid which might be assimilated in this manner.

According to Berthier 1000 lbs. of dry fir wood yielded 4 lbs. of ashes. In every 100 lbs. of these ashes, after subtracting the chloride of potassium and sulphate of potass, there remained 53 lbs. of basic oxide, consisting of salts of potash, soda, lime, magnesia, iron, and manganese.

Now 40,000 square feet, Hessian measure, of woodland yielded annually, according to Heyer, upon an average, 2650 lbs., Hessian, of dry fir-wood, which contain 56 lbs., Hessian, of metallic oxides. But, according to the experience of Malaguti and Sprengel, 1 lb., Hessian, of lime combines chemically with 10·9 lbs., Hessian, of humic acid; 5·6 lbs. of the metallic oxides would accordingly introduce into the trees 61 lbs., Hessian, of humic acid, which, admitting humic acid to contain 58 per cent. of carbon, would correspond to 91 lbs., Hessian, of dry wood. Hence there is only 91 lbs. which can be accounted for as possibly deriving its origin from the humus, and 2559 lbs. which must have proceeded from a different source.

There is another possible source, however, of humus, viz., through the agency of rain-water, which Liebig carefully examines. The quantity of rain which falls at Erfurt is 17½ lbs., Hessian, over every square foot of surface. If the whole of the water were absorbed by the roots of plants saturated with humate of lime, the plants growing on 40,000 square feet and receiving 700,000 lbs. of rain-water, would not take up more than 300 lbs. of humic acid, since one part of humate of lime is soluble in 2500 parts of water. But the amount of land mentioned produces 2580 lbs. of corn, in grain and straw alone. Hence the 300 lbs. of humic acid will not account for the quantity of carbon contained in the roots and leaves alone. Again, 100 parts of straw dried in air contain 38 per cent. of carbon: hence 1780 lbs. of straw contain 676 lbs. of carbon. In 100 parts of corn there are 43 parts of carbon; 800 lbs. must hence contain 344 lbs., that is, altogether, 1020 lbs. of carbon. By comparing these with other facts Liebig concludes that equal surfaces of land adapted for agriculture produce equal quantities of carbon.

The soil being thus repudiated as an element in accounting for the fruitful origin of carbon, we are obliged to recur to the only other possible source from which it can be derived, viz., the atmosphere. The mean of the experiments of Saussure, made at various seasons, leads to the inference that the quantity of carbonic acid in the atmosphere amounts to one thousandth of its weight. Now, as far as can be discovered by the analysis of airs from ancient jars and pyramids, the quantity of oxygen has been constantly the same in the atmosphere for thousands of years. We have no reason to believe that the relative proportion of carbonic acid in the atmosphere has increased during the same period. Hence it is possible that the oxygen stands in some fixed relation to it, that is to say, that two causes exist, one of which prevents the accumulation of carbonic acid by removing it as it is generated, and another which supplies oxygen in place of that which is lost by respiration, putrefaction, and combustion. These two causes are found united, Liebig considers, in the *vital action* of plants. If we take the sprig of a plant and introduce it into water, saturated with carbonic acid, we shall find that the whole of the carbonic acid disappears, and is replaced by oxygen, which may be collected and examined, the latter occupying exactly the same bulk as the former. This is a beautiful provision, as it tends directly to the preservation of a pure atmosphere for animal life. From the circumstance of the oxygen given out by plants being equal in bulk to the carbonic acid taken up, Liebig concludes that the carbonic acid is absorbed by the assimilating organs of the plant, where it is decomposed—the carbon being retained and the oxygen displaced. But it may be asked, is it possible that a body existing to the amount of only one thousandth part in the atmosphere, can supply the whole of such an essential ingredient of all the living masses of plants and animals which cover the surface of the earth ? The simplest use of arithmetic will soon demonstrate the possibility of this apparent paradox. Upon every Hessian square foot of the surface of the earth there rests a column of air, weighing 2216·66lbs., Hessian measure. Now the diameter, and consequently the surface, of the earth being known, the weight of the whole atmosphere may be calculated with the greatest accuracy. The thousandth part of this is carbonic acid, which contains twenty-seven per cent. of carbon. From which it results that the atmosphere contains 3000 billions of Hessian lbs. of carbon—a quantity which is certainly greater than the weight of all the plants, coals, and lignites upon the face of the earth.

It has been observed that during the night plants exhale carbonic acid, and absorb oxygen. The bulk of the latter however is never equal to the former, and hence Liebig concludes that, on the departure of light, the carbonic acid ceases to be decomposed, and being absorbed along with the juices, it is given out by the leaves, in quantity corresponding to that of the water which evaporates. From the absence of a proper explanation of the latter phenomena, some physiologists have considered the theory of the assimilation of carbon from the air as refuted, although the original experimenters had come to the conclusion which has just been developed. The reason of their erroneous inference has been powerfully portrayed by Liebig :

"All the talent and labour of botanists have been expended in reseaches on the structure and external form of plants, without calling into their assistance the resources of chemistry and physics, although calculated to explain the most simple phenomena, and powerfully adapted to discover the truth. The experiments and laws of these two sciences have always been neglected—they have been neglected because they have not been studied. All discoveries in physics and in chemistry, all explanations of chemists, must remain without fruit and be useless, because even to the great leaders in physiology, carbonic acid, ammonia, acids and bases, are sounds without meaning, words without sense, terms of an uuknown language which awaken no thoughts and no associations. They treat these sciences like the vulgar, who despise a foreign language in exact proportion to their ignorance of it; since even when they have had some acquaintance with them, they have not understood their spirit and application. Physiologists reject the aid of chemistry, in their enquiries into the secrets of vitality, although it alone could guide them in the true path: they reject chemistry, because in the pursuit of knowledge it destroys the subjects of its investigation, but they forget that the knife of the anatomist must dismember the body and destroy its organs, if an account is to be given of their form, structure, and functions."

Since humus then is not the source of the carbon of plants, for what purpose, it may be asked, are its decomposing elements destined ? Liebig answers this question in a manner if not demonstrative, at least highly plausible. Humus, he observes, nourishes plants not by being absorbed and assimilated *per se*, but by presenting to their roots in a decomposing state a slow and continual source of nourishment, in the form of carbonic acid. This source is only beneficial to plants until their leaves are formed, which enable them to assimilate carbon from the atmosphere.

*Assimilation of hydrogen.* The solid portion of plants or lignin contains carbon and the constituents of water, or the elements of carbonic acid and a certain quantity of hydrogen : 100 parts of carbonic acid require the addition of 8·04 parts of hydrogen to form lignin, and 72·35 parts of oxygen are evolved in the state of gas, if we suppose that the hydrogen is derived from the decomposition of water—so that, as in the case of the assimilation of carbon, by the decomposition of carbonic acid, we may consider that the assimilation of hydrogen is preceded by the decomposition of water.

*Assimilation of nitrogen or azote.* We have seen that carbon and hydrogen are not presented in their simple state to the absorbing vessels of plants, but are taken up in union with oxygen; *pari passu*, we might infer that azote would not gain admission in the uncombined form in which it exists in the atmosphere. That the azote is derived from the atmosphere and not from the soil is obvious from the following considerations. Let us conceive a farm properly cultivated, and of sufficient size to be supported on its own resources: a certain quantity of azote will then exist upon it, in the form of men, animals, cattle, corn, fruits, and excrementitious matter; the farm is managed without the addition, from extraneous sources, of azote in any form. The products are annually exchanged for money and other necessaries of life, which contain no azote, but by exporting a certain portion of the corn and cattle, a certain quantity of azote is removed without being replaced. Yet after the lapse of some years the amount of azote has increased. Hence it is evident that plants, and consequently animals, must derive their azote from the atmosphere. Liebig discusses the condition of the azote as it is presented to plants in a very able manner, and has adduced several novel experimental results. Ammonia, he concludes, is the form in which *nutritive azote*, if we may use the expression, exists in the atmosphere. From whence proceeds the immense depôt of ammonia requisite for this purpose?

The ultimate products of the decomposition of animal substance are exhibited in two different forms: in cold or temperate climates, under the form of ammonia; in the torrid zone, under that of nitric acid. However, it has been demonstrated that the formation of nitric acid is constantly preceded by the production of ammonia: the latter is the final product of the putrefaction of animal matter; the nitric acid results from the slow combustion of this product.

A generation of a thousand millions of men is renewed every thirty years; millions of animals perish and are reproduced in a shorter period: what becomes of the azote with which these bodies were so largely endowed? It has been dissipated in the air by the law of gaseous diffusion through the regions of the atmosphere. That ammonia is present in the air is proved by the fact of its presence in rain water: this is easily rendered obvious to the senses by adding a little sulphuric or muriatic acids to a quantity of rain water and evaporating to dryness: these acids combine with the ammonia, and deprive it of its volatility—the residue then contains sal ammoniac or sulphate of ammonia, whose presence may be detected by the aid of bichloride of platinum, and still more readily by the odour developed on the addition of pulverized hydrate of lime: ammonia also exists in snow-water, and in this case, when the odour is evolved, it possesses a strong taint of perspiration and excrementitious matter, which sufficiently points to its animal origin. This investigation has elicited an explanation of a fact, which must have struck those engaged in the minute details of pharmacy; namely, the precipitation produced in distilled water by subacetate of lead. According to Liebig, this proceeds from the presence of carbonate of ammonia: it may be obviated by the addition of a small portion of alum or sulphuric acid before commencing the distillation: the softness of rain-water is also due to the same cause. A portion of the ammonia dissipated in the atmosphere gains admission to the soil by means of rain-water, some of this evaporates again with the water, while another portion is absorbed by the roots, and, being assimilated, gives origin to albumen, gluten, &c.

Liebig shows that the action of dung is to supply ammonia to the roots of plants for the assimilation of azote. In Flanders, putrid urine is successfully employed as manure: every one knows that the characteristic feature in this state of the fluid is the evolution of ammonia: the *guano* of Peru, or dung of birds, is valuable from the same cause. The influence of lime in the soil is now manifest from the facts detailed. The carbonate of ammonia dissolved in the rain-water decomposes the sulphate in the same manner as in the manufactories of sal ammoniac. Ferruginous soils owe their value to the property of absorbing ammonia: carbon, which absorbs ammonia most powerfully, is highly useful for the same reason.

*Inorganic constituents of Plants.* No one can suppose that the acid constituents of plants are fortuitous, or that plants contain more acid than is required for their existence; hence we should expect that oxides would be necessary for vegetation, because all acids which plants contain are found in the state of neutral or acid salts. Saussure and Berthier analyzed pines from Breven and La Salle, and concluded that different soils exercise an action upon the proportion of the metallic oxides present; but what is curious, and these chemists did not observe it, the saturating power of the oxides is almost identical in the two cases. This explains some important considerations in pharmacy; for most of the species of opium contain meconic acid in combination with extremely variable quantities of narcotine, codein, morphia, &c.; but the quantity of one of these bases always increases as the other diminishes: the smallest quantities of morphine are found constantly accompanied by a maximum of narcotine; the presence of alkalies, therefore, appears necessary for the development of some, and the action of particular salts is indispensable for the existence of other plants. Some have supposed these saline matters to be generated by the vital action of plants; but when we are acquainted with the facts, that common salt is evaporated into the air along with sea-water, and that saltpetre is dissipated by the action of heat without being decomposed, we can at once detect the fallacy of such deductions. From the previous considerations, it may therefore be concluded, according to Liebig, that carbonic acid, ammonia, and water, are the indispensable sources of the food of plants; but that for their perfect development, and in order to afford facilities for the operation of these elements, the presence of inorganic substances is also undoubtedly necessary.

Highly plausible though the view be which ascribes the source of vegetable azote to the decomposition of ammonia, we may ask in the language of the Hottentot preacher, and after the example of Liebig himself in reference to humus—whence came the primitive ammonia? Liebig is of opinion that the ammonia, if evolved along with boracic acid in volcanic situations, is an original constituent of the earth; but the quantity and sources are too limited to afford sufficient supply for such purposes as we have discussed. This point may therefore be considered as still *sub judice.*

*Poisons, miasms, and contagions.* Inorganic substances when introduced into the animal system act in the first instance chemically, and by producing abnormal action upon the nervous system give origin to the symptoms of poisoning, such as irritability of the stomach and paralysis. The iodide of potassium, sulphocyanuret of potassium, ferrocyanuret of potassium, nitrate, chlorate, silicate of potass, and in general salts with an alkaline base, pass without alteration into the blood, the sweat, the chyle, and the bile, and are thrown out by the kidneys. The citrates, the tartrates, and acetates of alkaline bases, in their circulation through the system, on the other hand, are converted into carbonates ; this fact proves that they have accumulated in their passage a quantity of oxygen. In order to convert one atom of acetate of potash into carbonate, 8 atoms of oxygen are required, 2 or 4 remaining in combination with the alkali, and the remainder being evolved as free carbonic acid. During the circulation of these salts through the lungs, Liebig conceives that these acids take part in the peculiar process for which these organs are set apart; a certain quantity of the oxygen gas inspired unites with their elements, transforming their hydrogen into water, and their carbon into carbonic acid. A similar effect is produced when impure solutions of these salts are left in contact with the atmosphere; so that we have a mode of similating respiration artificially; on the other hand, mineral acids, and fixed vegetable acids, and mineral salts with an alkaline base, prevent *decay.*

Liebig has shown that the decay of organic matter, or *eremacausis* (slow combustion), as he terms it, is similar to the action which takes place in the lungs. This action would be arrested if an excess of acids or metallic salts could reach the lungs; but the accumulation of the latter is prevented by the circumstance that membranes are not permeated by concentrated saline solutions. A dry bladder remains more or less dry in a saturated solution of common salt ; fresh meat which has been strewed with salt will be found, after twenty-four hours, to be swimming in brine, although no water has been added ; the water in immediate contact with the salt has dissolved the latter, and consequently lost the power of permeating animal substances ; hence the mode by which salt preserves meat, viz. by extracting the water from it. The same observation pertains to alcohol. Liebig applies these facts to explain the action of salts when introduced into the alimentary canal; a concentrated saline solution when conveyed into the stomach, by extracting water from the coats of that organ and the intestines, produces thirst and a purgative action; a diluted solution, on the contrary, is absorbed. That this is the primary action of purgatives is abundantly obvious from the fact that all mineral salts without alkaline bases, have the same action ; it matters not what the base or acid is, whether potash, soda, or magnesia, or phosphoric, sulphuric, nitric, or hydrochloric acids.

The salts of metallic oxides possess a tonic action. Instead of abstracting matter from the tissues they unite with them, (C. G. Mitscherlich and Dr. R. D. Thomson,) they constitute the true inorganic poisons ; they are never in the excretions. When arsenious acid is given in solution, it may enter into the blood and poison it, that is, combine with the fibrine in solution, and with every blood-globule with which it comes in contact. But how, it may be asked, can we suppose a grain of arsenic, which has been recorded as having produced death, to occasion such a chemical change in the blood, a fluid containing such a comparatively prodigious quantity of matter capable of combining with the arsenic? The contrast however will not appear so remarkable, when we consider that 1240 grains of arsenious acid require for combination 6361 grains of fibrine; now we could form an artificial fluid similar to the blood, if we were to dissolve 100 parts of dry fibrine in 30,000 parts of water; so simple is the dissipation of mystery, when we remove all error and wrestle with the pure truth. With respect to the action of strychnine, other alkalies, and prussic acid, it is true that no primary action has hitherto been elicited, but ignorance is no argument against the existence of anything; the reason for supposing that no physical change takes place, because the action is so exceedingly rapid, is fallacious, especially since the experiments of Tiedemann were published, (Brit. and For. Med. Rev. vol. I. p. 243.) He injected an ounce of spirit of wine into a vein in the thigh of a middle-sized dog: the injection was scarcely over, before the odour of the alcohol was perceptible in the expired air; again no poison acts like a blow on the brain. Hydrocyanic acid is perhaps the most rapid of all poisons in its action, and yet after numerous observations we have never seen it exhibit any symptom upon the animal economy earlier than fifteen seconds, a period sufficiently protracted to enable it, according to the results of the German physiologists, to pass through the whole sanguineous circulation.

The substances to which we have hitherto referred exercise their influence upon the animal economy, by forming chemical combinations, or by containing a poisonous principle; but we are acquainted with another class of bodies, not less mortal, which act by virtue of the condition in which they are found. To comprehend the mode of operation, it is necessary to understand the processes of putrefaction, fermentation, and *eremacausis.* The term fermentation is, in common language, applied to that change which occurs in bodies, where gaseous bodies destitute of odour are disengaged: putrefaction denotes that spontaneous decomposition of organic substances, in which gases with a disagreeable smell are emitted. Chemistry demonstrates, however, that both of these processes are of a similar nature, the one implying the decomposition of substances destitute of nitrogen, the other of substances which contain it. Eremacausis differs from the two processes mentioned, in the circumstance that it cannot take place without the access of air: we have an example of it afforded in the decay of woody fibre: when this substance is exposed to the action of air or oxygen, the latter is converted into an equal volume of carbonic acid, and when the oxygen disappears the decay ceases. If the carbonic acid formed be replaced by oxygen, the decay recommences: hence this decay is precisely similar in its results to the combustion of pure carbon at very elevated temperatures; according to Liebig this is the process which takes place in the lungs during respiration.

But to return to fermentation: according to a law proposed by La Place and Berthollet, "a molecule set in motion by any power can impart its own motion to another molecule with which it may be in contact." It is a mechanical law which is brought into force whenever the resistance which is opposed to motion is not sufficient to neutralize it. Now yeast is a decomposing body, the molecules of which are in a condition of motion or in the act of losing their equilibrium. If agitated in a vessel filled with a solution of sugar, the molecules of yeast communicate their condition to the particles of the sugar; the result is the formation of carbonic acid and alcohol, compounds in which the constituents are retained in combination with a greater force than in sugar: when the substance made to act on sugar is in a different state of decomposition, different products are the result: thus when brought in contact with rennet or putrefying vegetable juices, it is converted into lactic acid, mannite, and gum; under the condition we have mentioned, that is in contact with sugar, yeast disappears and none is reproduced; but when added to gluten contained in vegetable juices, new yeast is formed; yeast therefore is produced from gluten. Hence it follows that a decomposiug body added to a mixture in which its component parts exist, is capable of reproducing itself in that fluid, in the same manner as new yeast is formed when yeast is added to liquors containing gluten.

These principles, it is obvious, may be applied to organic substances which are products of the animal economy. We know that all elements of these substances are derived from the blood, the most complex of all existing substances. Other bodies can act upon the blood, can attract and change it: but it possesses within itself a power of transformation. When putrid matter is laid upon fresh wounds, as in dissecting, the blood is poisoned, and direful effects result. The poison of decayed sausages belongs to this class of poisons. Several hundred cases of death have occurred from the use of this food. These sausages consist of liver, blood, &c. mixed with salt and spices, which are placed in bladders, or intestines, and after being boiled are hung up to be smoked. When these are properly prepared they may be preserved for months; but when the quantity of condiments is deficient they undergo a kind of putrefaction which commences in the centre of the sausage. No gas appears to escape; but they become paler in colour, and more soft and greasy in the putrefied parts, and they are found to contain lactic acid and lactate of ammonia. The cause of this action has been variously attributed to different sources. Prussic acid, sebacic acid, and benzoic acid have all been blamed for the mischief; but these causes are the dreams of theorists, not the deductions of practical men. The action elicited is situated in the function of assimilation, if we may judge from the aspect of the patients affected. The muscular development disappears, with all similarly constituted parts of the body; the patient becomes dried up like a mummy; the saliva is viscid and stinking; the dead body presents the appearance of coagulation, and does not putrefy. The result is analogous to the action of alcohol and boiling water. It is scarcely possible to mistake the action of the poison now described; for the experiments of Colin have distinctly proved that muscular fibre, urine, cheese in a state of decay can communicate a decomposing influence to much simpler bodies: when mixed with sugar they produce fermentation. Liebig contends that when fat and putrefying muscle produce death by being placed on living wounds, they act in a similar manner. They communicate their own state of decomposition to the blood *from which they*

*were themselves formed.* Poisons, such as those of smallpox, syphilis, plague, which are formed from the blood, are capable of reproducing themselves when introduced into that fluid.

The similarity of the phenomena induced by contagions and by yeast is so striking that it attracted the attention of Hippocrates; but to that distinguished physician the idea was merely figurative. He could have no tangible notion of the nature of the action, because he was ignorant of the chemical influence of yeast itself. Between the mode of action of yeast and contagion there appears to be almost as strong an analogy established as between lightning and electricity. Contagious matters are neutralized, Liebig states, by heat and alcohol, while acids, mercurial salts, sulphurous acid, chlorine, iodine, bromine, aromatic principles, volatile oils, empyrematic oils, smoke, coffee, deprive them of their contagious powers. The same influences, without exception, retard in general fermentation, eremacausis, and putrefaction. Contagious matter, we thus discover, must be a solid organized substance, capable of reproducing itself in the fluid from which it has been formed. By this consideration we shall be enabled to appreciate, at least in part, some of the poetical philosophy of Jahn. (System der Physiatrik, 8vo, 1835; Br. and For. Med. Rev., vol. IV., p. 120.)

The inferences which we deduce from the views of Liebig are opposed *in toto* to the idea of inorganic gaseous substances being capable of producing those diseases which possess regular phases. The average of the phenomena of smallpox, measles, &c. are not less regular than the phases of the heavenly bodies. Interruptions or irregularities, it is true, we often find recorded. But how often do these result from errors of observation, or from influences whose value is capable of calculation? These views are also opposed to the animalcular or infusorial theory of diseases, which is certainly possessed at least of great ingenuity. It assumes the theory of fermentation broached by Cagnard Latour, that the process is occasioned by the operation of infusoria—a gratuitous assumption, according to Liebig. It would admit of less objection if the animalcules are considered to be in a state of decomposition when they reach the circulation. In this case it is obvious that the theory tends to explain nothing, save that the occurrence of swarms of putrefying animalcules in the air might account for epidemics. If we admit their decomposition we might as readily grant the presence of volatilized putrefying matter derived from various sources.

It has been well observed that "the condition of medical science requires the separation of what is ascertained from what is only imagined." (Dr. Marshall Hall.) Now, much mystery has been thrown over many diseases by the substitution of *words* for *ideas*. What definite notion can we affix to the terms dynamic power of the organism in the present state of our knowledge? We have only the means of studying the effects of vital influence: it is beyond our power to ascend by pure induction from the effects to their causes. Many escape from their difficulties by adopting, with Dr. Prout, the old hypothesis of the existence of *independent* vital principles or agents superior to and capable of controlling and directing the agents operating in inorganic matters, on the presence and influence of which the phenomena of organization and life are supposed to depend. The chemical forces acting in the system, according to them, are subject to this invisible cause. Of the existence of this cause we are only made aware by the phenomena which it produces. Its laws must be investigated just in the same manner as we examine those of the other powers which effect motion and changes in matter. When a chemical compound is introduced into the stomach it must exercise a chemical action upon every body with which it comes in contact. It is opposed to the vital power. If the latter predominate the substance is digested. If the chemical compound resists the vital power, its operation is *medicinal* or *poisonous*, according to the ascendancy which it attains. All the phenomena with which we are acquainted in relation to contagions are opposed to the idea of their possessing life. Their effects, it is true, have some resemblance to some actions of the animal economy. But the cause of these effects is simply a chemical action, capable of neutralizing other chemical actions exercised in a contrary direction. Some of the poisons generated in the body by disease lose their power when introduced into the stomach, while others retain their influence. The poison of the contagious fever in cattle, and small-pox matter, which are alkaline or neutral, are destroyed by the action of the acid in the stomach; while the poison of sausages, which is acid, has its power increased, or, at least, its energy is not retarded by the same influence. Diseases, however, are not contagious unless the matter which produces the disease meets with another substance by means of which it can be reproduced. Saliva is capable of converting sugar into lactic acid, without the addition or loss of any element. But in this process the exciting body is not reproduced, for the conditions necessary to its reproduction do not exist in the elements of the sugar. But when yeast excites fermentation in a mixture of sugar and gluten, the former is reproduced, because gluten is present from which the yeast had been primarily formed. Hence the formation of the existing body depends: 1, on the presence of the substance from which it was originally formed; 2, on the presence of a second substance, which is susceptible of decomposition in contact with the exciting body. Now in applying this reasoning to the action of contagion, we shall find that we must admit the presence of a second body in the blood capable of being decomposed. So long as no contagious matter gains access to this substance the human body is healthy, although in a state of *predisposition* to be affected. By this view we can account for the fact of many individuals escaping from the action of contagious diseases during the prevalence of epidemics. The second substance may be produced by imperfect assimilation, by a vitiating diet, and by a variety of causes which have hitherto been entirely overlooked. We shall now be enabled to estimate the value of attention to diet in warding off disease, and shall discover that this department of the physician's knowledge is even of superior importance to his acquaintance with therapeutics. We shall also feel the necessity in our practice of substituting wholesome plain food for the unnatural and often putrefying aliment contrived by the vitiated and pampered taste of dyspeptic man.

Liebig likens the action of cowpox virus to that of low yeast. It communicates its own state of decomposition to a matter in the blood, and from a second substance is itself reformed by a different mode of production. The product possesses the mild form and all the qualities of cowpox lymph. The predisposition to infection by smallpox must cease after vaccination, because the cause of the predisposition has been removed by the action of the vaccine lymph. But the predisposing substance may be again generated in the same individual, so that he may again become liable to the contagion, and a second or a third vaccination may be required to remove the regenerated susceptibility. From the preceding considerations it would appear that when matter undergoing decomposition is the product of a disease it is called *contagion.*

We now turn to another cause of disease, viz. *miasm*, which is the product of the decay or putrefaction of animal and vegetable substances. Contagion or gaseous contagious matter occasions disease by reproducing itself in the blood. *Miasm* produces disease, but is not regenerated in the blood. All observations upon gaseous contagions show that they are substances in a state of decomposition. Air charged with them deposits upon vessels filled with ice a fluid, which soon becomes turbid and putrefies. All gases exhaled from putrefying matter have a bad smell, proceeding from substances in a state of decomposition, which is volatilized along with the gases. A number of metals, &c., exhale an odour when rubbed, as arsenic, phosphorus, &c., but only when in a state of eremacausis, that is to say, when they are oxidized at the ordinary temperatures. These gases are generally accompanied with ammonia, which is frequently the cause of their assuming the gaseous form. It may be detected in the air of a contagious room by condensing the moisture of the air by ice, in the manner described, and adding corrosive sublimate. Liebig accounts for the action of acids in destroying contagion, by their neutralizing power upon the ammonia, upon the presence of which the volatile nature of the contagion depends. Muriatic and acetic acids are best adapted for this purpose. Chlorine possesses similar powers; but, in consequence of its direful action upon the human economy, its influence where human beings are breathing is to be carefully shunned.

Such is a brief outline of the new views of Professor Liebig. We trust he will continue to pursue his researches, and render doubly sure many of his statements, which may be hesitatingly received, more from their novelty than from any apparent want of consonance to nature. We cannot, however, conclude without expressing our approbation of the admirable manner in which the English version has been executed, and our reprobation of the extravagant price of the volume. The French edition may be had gratuitously by the purchasers of the first volume of the *Chimie Organique.*

